# Two-Stage Semi-Continuous 2-Keto-Gluconic Acid (2KGA) Production by *Pseudomonas plecoglossicida* JUIM01 From Rice Starch Hydrolyzate

**DOI:** 10.3389/fbioe.2020.00120

**Published:** 2020-02-28

**Authors:** Lei Sun, Da-Ming Wang, Wen-Jing Sun, Feng-Jie Cui, Jin-Song Gong, Xiao-Mei Zhang, Jin-Song Shi, Zheng-Hong Xu

**Affiliations:** ^1^The Key Laboratory of Industrial Biotechnology, National Engineering Laboratory for Cereal Fermentation Technology, School of Biotechnology, Jiangnan University, Ministry of Education, Wuxi, China; ^2^School of Food and Biological Engineering, Jiangsu University, Zhenjiang, China

**Keywords:** 2-Keto-gluconic acid, *Pseudomonas plecoglossicida*, two-stage semi-continuous fermentation, rice starch hydrolyzate, fermentation performance

## Abstract

A two-stage semi-continuous strategy for producing 2-keto-gluconic acid (2KGA) by *Pseudomonas plecoglossicida* JUIM01 from rice starch hydrolyzate (RSH) has been developed. The initial glucose concentration (140 g/L) was selected for first-stage fermentation due to its highest 2KGA productivity of 7.58 g/(L⋅h), cell weight of 3.91 g/L, and residual glucose concentration of 25.00 g/L. Followed by removing 70.0% (v/v) of the first-stage broth and feeding 400.0 g/L of glucose to the second-stage fermentor, a total of 50680.0 g glucose was consumed, and 50005.20 g 2KGA was obtained with a yield of 0.9867 g/g by *P. plecoglossicida* JUIM01 after a 3-cycle two-stage semi-continuous fermentation. Our results indicated that the developed two-stage semi-continuous fermentation could be industrially applied due to its high 2KGA concentration, 2KGA yield and operation efficiency.

## Introduction

Erythorbic acid, also known as D-isoascorbic acid, is a stereoisomer of ascorbic acid. Its global market demand is approximately 40,000 tons/year due to its significant applications in preventing food oxidation, inhibiting the decrease of color, flavors and aroma, and blocking the formation of carcinogenic ammonium nitrite during food processing (Walker, 1991; Wei et al., 2008). Erythorbic acid and its salts are generally recognized as safe (GRAS) by the US Food and Drug Administration (FDA) and approved as food ingredients E315 (free acid) and E316 (sodium salt) in Europe with no safety concern (Aguilar et al., 2016). Recently, erythorbic acid and its salts have seen their applications extended to include roles in mushroom post-harvesting as an oxygen scavenger, in the oil and gas industry as an oxygen scavenger, in feed industries as a calcium-rich additive and as acidulates, and in the florescent materials field to promote the synthesis of CdS quantum dots (Liang et al., 2014; Joven et al., 2015; Al Helal et al., 2018; Ping et al., 2018).

2-Keto-D-gluconic acid (2KGA) is a key intermediate for erythorbic acid and erythorbate production. It is also a versatile chemical applied in the cosmetics and pharmaceutical industries (Li et al., 2016). For 2KGA preparation, at least three methods including chemical synthesis, enzymatic conversion, and microbial fermentation have been proposed. However, chemical and enzymatic methods had limits to large-scale production due to their critical reaction conditions, low yield and/or high process costs (Elseviers et al., 2000; Tanimura et al., 2003). Microbial fermentation is the main industrial 2KGA production method with two consecutive oxidations by glucose dehydrogenase and gluconate dehydrogenase.

$$\text{Glucose}\overset{\text{Glucose-1-dehydrogenase}}{\to }\;\text{Gluconic}\;\text{Acid}\;\overset{\text{Gluconate dehydrogenase}}{\to }\;2-\text{keto−Gluconic Acid}$$

*Gluconobacter*, *Pseudogluconobacter*, *Pseudomonas*, *Serratia*, and *Klebsiella* are the potential genera for 2KGA production with various fermentation performances. For example, *Serratia marcescens* NRRL B-486 yielded 2KGA ranging from 0.85 g/g to 1.0 g/g glucose using continuous fermentation (Minisenhermer et al., 1965). Since 1998, our group has screened several 2KGA producing strains, such as *Pseudomonas fluorescens* AR4, *Arthrobacter globiformis* C224, and *Pseudomonas plecoglossicida* JUIM01. Among them, *P. fluorescens* AR4 showed a high substrate tolerance (>120 g/L glucose) and a low susceptibility to phage infection (Sun et al., 2006). Other engineered strains have also been constructed by overexpressing the *ga2dh* gene of *Gluconobacter oxidans* which produced approximately 486 g/L 2KGA from 480 g/L gluconic acid (Shi et al., 2015).

In order to overcome the inhibitory effects of substrate and/or product in the conventional batch processes, various culture systems, e.g., fed-batch, semi-continuous and continuous fermentation, have been developed. Three-cycle DO-stat repeated fed batch fermentation was developed to yield 72 g of 2KGA from 110 g of cassava by the immobilized *Pseudomonas aeruginosa* with a maximum productivity and cell concentration of 0.55 g/(L⋅h) and 35 g/L, respectively (Chia et al., 2008). Our group has also proposed a semi-continuous process, continuous process and direct conversion of glucose to 2KGA by *P. fluorescens* AR4 or *A. globiformis* C224 in an unsterile and buffer-free system with the purpose to increase its final concentration and improve the handling process (Teng et al., 2013; Sun et al., 2015). A production of 195 g/L 2KGA, 3.05 g/(L⋅h) productivity and a yield of 1.07 g/g were achieved in a non-sterile and buffer-free system by *P. fluorescens* AR4 (Sun et al., 2015). Our previous results also showed that the semi-continuous fermentation of *P. fluorescens* AR4 was able to produce 444.96 g/L of 2KGA, with a productivity of 6.74 g/L⋅h and a yield of 0.93 g/g, the values of which reached the industrially acceptable levels for 2KGA production (Sun et al., 2012). However, the industrial application of the 2KGA semi-continuous process highlighted some disadvantages including a lot of time spent on equipment sterilization to avoid contamination after reactor turnover. Recently, a two-stage fed-batch operation was proven to favor a significant increase of DHA threshold values by culturing the viable microbial cells in the first stage and converting a high concentration of glycerol to DHA in the second stage (Bauer et al., 2005). The present study therefore aimed to develop a two−stage semi-continuous fermentation system with the potential to improve the industrial 2KGA fermentation performance by *P. plecoglossicida* JUIM01, through improving its working efficiency and increasing the utilization ratio of equipment with the aim to substitute the current 2KGA batch or semi-continuous production process.

## Materials and Methods

### Microorganism and Media

*Pseudomonas plecoglossicida* JUIM01 was screened and stocked in our laboratory (Wang et al., 2018; Sun et al., 2019). The media for the stocking strain consisted of (g/L): peptone 10.0, NaCl 5.0, beef extract 5.0 and agar 20.0. Media for preparing the seed contained (g/L, pH 7.0): glucose 20.0, urea 2.0, corn syrup powder 5.0, MgSO_4_⋅7H_2_O 0.5 and KH_2_PO_4_ 2.0.

The concentrated rice starch hydrolyzate (RSH) containing 450 g/L of glucose and 3.5 g/L of protein was prepared by Parchn Sodium Isovitamin C Co. Ltd (Dexing, Jiangxi, China), and diluted to specific glucose concentrations for two-stage fermentation. Media for the first-stage fermentation contained (g/L, pH 6.7): glucose 140.0, corn syrup powder 7.5, CaCO_3_ 39.0. Glyceryl polyether at 0.02% (*v/v*) was added as defoamer. The feeding media for the first-stage was (g/L, pH 6.7): glucose 140.0, corn syrup powder 7.5, urea 0.15, KH_2_PO_4_ 0.1 and CaCO_3_ 39.0. The feeding media for the second-stage was (g/L, pH 6.7): glucose 300.0 and CaCO_3_ 82.5.

### Cultivation and Two-Stage Fermentation

The seed of *P. plecoglossicida* JUIM01 used for 50-L scale fermentation was prepared by activating the stock cells in 50 mL of seed media at 30°C for 20 h and cultivating the activated cells in a 5-L fermentor with working a volume of 3.5 L. The agitation speed and aeration rate of the 5-L fermentor (Green Bio-engineering Co., Ltd, Zhenjiang, China) were set as 400 rpm and 105.0 L/h, respectively. After a 24-h cultivation with a cell concentration of 8.05 g/L (approximate OD_650__nm_ of 14.0), the culture was used as an inoculum for two-stage semi-continuous fermentation.

**FIGURE 1:**
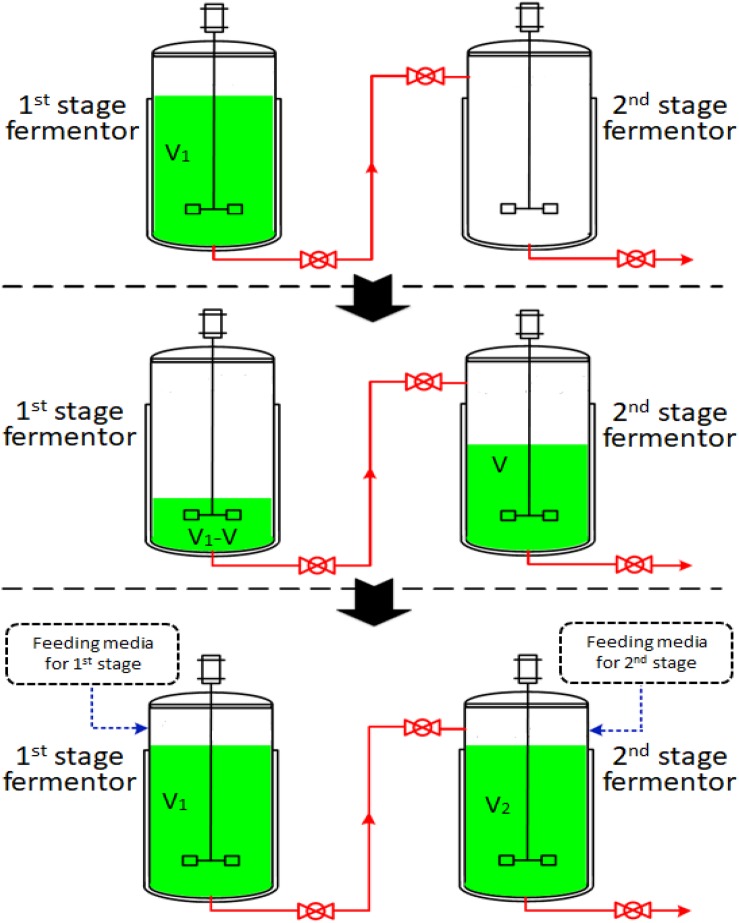
A schematic diagram of a two-stage semi-continuous fermentation system for 2KGA production by *P. plecoglossicida* JUIM01 (V of first-stage cultivated broth in fermentor 1 was transferred to fermentor 2 as the inoculator. The feeding media (V and V_1_-V) were fed into first- and second- stage fermentors, respectively. The second-stage cultivation proceeded until the glucose to be consumed was below 1.0 g/L).

The two-stage semi-continuous process consisted of at least two 50-L mechanical stirred fermentors (Green Bio-engineering Co., Ltd, Zhenjiang, China). Temperature, DO concentration, pH, agitation speed and airflow rate were monitored on-line. The operational procedures were outlined in Figure 1. The first-stage fermentation started with the inoculation of the seeds into 35 L of media (V_1_) and this was cultivated at 30°C with an aeration rate of 42.0 L/min and an agitation speed of 450 rpm under a pressure of 0.03 MPa. The first-stage cultivated broth was taken out to be used as the seed in the second-stage fermentor after a specific cultivation time (the time at which residual glucose concentration had decreased to specified levels). The feeding media of the first- and second-stages were fed into first- and second-stage fermentors with volumes of V and V_2_-V (V_2_ = 35.0 L), respectively. The first-stage fermentor maintained cultivation and was used as the seed for the next run. The second-stage fermentors were cultivated at 33°C, with an aeration rate of 42.0 L/min and an agitation speed of 450 rpm under a pressure of 0.03 MPa. Each second-stage fermentation ended when glucose concentration was below 1.0 g/L. Samples were withdrawn every four hours to determine the concentration of residual glucose, concentration of 2KGA, cell concentration and pH.

### Analytical Methods

Cell growth was expressed as OD_650__nm_ (optical density at 650 nm) or dry cell weight (DCW) (OD_650__*nm*_ of 1.0 was equivalent to 0.575 g DCW/L). The culture supernatant was obtained by centrifuging broth at 3,500 × *g* for 20 min, diluted with H_2_O and pH adjusted to 1.5 with 1 M HCl. The optical rotation degree and glucose concentration of the diluted supernatant were determined using a polarimeter (Precision Instrument Co., Ltd., Shanghai, China) and a biosensor analyzer (Shandong Academy of Sciences Institute of Biology, Jinan, China) at 25°C, respectively. 2KGA concentration was calculated with the following equation:

(1) Y=-0.88X+10.5275X2

X_1_ and X_2_ represent 2KGA and glucose concentrations (g/L), respectively. Y represents the optical rotation degree (°). Coefficients −0.88 and 0.5275 represent the optical rotation degree of 10 g/L of 2KGA and 10 g/L of glucose, respectively.

2-Keto-Gluconic Acid productivity was defined as the amount of 2KGA produced per hour per liter. 2KGA yield was calculated as the amount of 2KGA produced divided by the amount of glucose consumed. The glucose consumption ratio was calculated as the amount of consumed glucose divided by the initial amount of glucose.

### Statistical Analysis

All of the fermentations were performed in duplicate. One-way analysis of variance (ANOVA) and Duncan’s test were used for statistical analysis.

## Results and Discussion

### Effect of Glucose Concentration in First-Stage Fermentation on 2KGA Production Performance

Pure sugars and starch hydrolyzates have been widely used as the carbon sources for microbial organic acid production. Corn, wheat and rice starch are abundant inexpensive renewable resources which could reduce fermentation costs as substitutes for expensive pure sugars, however pure sugars give high final bioorganic acid concentrations and productivity. Our previous results have proven that RSHs showed similar 2KGA fermentation performances to those of pure glucose (Sun et al., 2012). Herein, on the basis of the previous study, a two-stage cultivation strategy was designed by cultivating a high density of *P. plecoglossicida* JUIM01 cells at a first-stage for 2KGA production during a second-stage. Glucose with concentrations of 80.0, 110.0, 140.0, 170.0, and 200.0 g/L was examined to determine its influence on the 2KGA production performance during the first-stage. As the summarized results in Table 1 show, an increase in glucose concentration from 80.0 to 200.0 g/L led to prolonged cultivation periods from 12.5 h to 32.5 h. Our previous results also proved that a high glucose concentration of 200.0 g/L increased the fermentation time of *P. fluorescens* AR4 to 36 h (Sun et al., 2012). Accordingly, an initial glucose concentration of 200.0 g/L resulted in the highest 2KGA production of 175.28 g/L. 2KGA yields of 0.9549, 0.9746, and 0.9678 g/g were obtained from the fermentation using 110.0, 140.0, and 170.0 g/L of glucose, respectively. On the basis of the 2KGA yield, productivity and cell concentration, we chose 140.0 g/L as the initial glucose concentration for the second-stage fermentation due to it producing the highest total 2KGA, productivity and cell concentration of 0.9746 g/g, 7.58 g/(L⋅h) and 3.91 g/L, respectively.

**TABLE 1:** Summary of 2KGA production from different glucose concentrations at first-stage fermentation by *P. plecoglossicida* JUIM01.

Initial glucose concentration (g/L)	Residual glucose concentration (g/L)	Maximum cell concentration (g/L)	2KGA concentration (g/L)	2KGA yield (g/g)	Fermentation time (h)	2KGA productivity (g/L⋅h)
80.0	0.12 ± 0.04	3.70 ± 0.09	70.79 ± 2.28	0.8849 ± 0.0285	12.5 ± 0.6	5.67 ± 0.15
110.0	0.15 ± 0.08	3.83 ± 0.16	105.04 ± 1.68	0.9549 ± 0.0152	16.5 ± 0.6	6.37 ± 0.14
140.0	0.13 ± 0.06	3.91 ± 0.13	136.44 ± 2.74	0.9746 ± 0.0196	18.0 ± 0.7	7.58 ± 0.18
170.0	0.17 ± 0.07	3.32 ± 0.12	164.53 ± 1.62	0.9678 ± 0.0095	26.0 ± 0.7	6.33 ± 0.10
200.0	1.98 ± 0.63	3.18 ± 0.11	175.28 ± 6.14	0.8764 ± 0.0307	32.5 ± 1.9	5.40 ± 0.16

### Fermentation Process at Glucose Concentration of 140 g/L in First-Stage Fermentation

**FIGURE 2:**
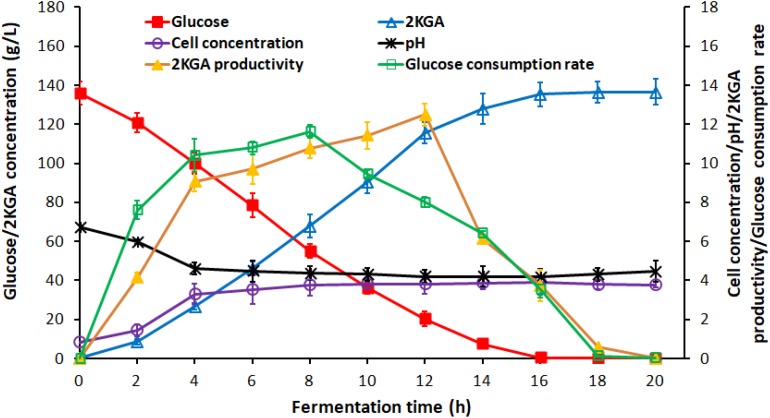
Time course of 2KGA production, residual glucose concentration, cell concentration and pH during batch production at first-stage fermentation by *P. plecoglossicida* JUIM01 with an initial glucose concentration of 140.0 g/L.

Generally, the residual glucose concentration from the previous stage in semi-continuous or cell recycled cultures, depending on the cultivation time, provides the activated cells with high densities for next stage without a lag phase. Figure 2 presented the time course of 2KGA batch fermentation by *P. plecoglossicida* JUIM01 at a glucose concentration of 140.0 g/L.

2-Keto-Gluconic Acid production and cell concentration increased rapidly to 26.76 g/L and 3.31 g/L, respectively, after the first 4-h fermentation. The exponential phase growth of *P. plecoglossicida* JUIM01 occurred from 4 h to 16 h with a rapid increase in 2KGA concentration to 135.29 g/L and cell concentration to 3.91 g/L. From 16 h to 24 h, fermentation entered a stationary phase with a maximum 2KGA concentration of 136.47 g/L and a gradual decrease of residual glucose to 0.10 g/L. Due to 2KGA accumulation, pH values decreased significantly from 6.75 to 4.61 and then remained at a relatively stable level of about 4.3. As for the two-stage cultivation, the optimal ending point for the first-stage was a cultivation time of 8∼10 h (residual glucose ranging from 20.00 to 55.00 g/L) with a high catalytic activity of *P. plecoglossicida* JUIM01 and 2KGA productivity of over 10.00 g/(L⋅h), which benefited the second-stage of fermentation.

### Effect of Residual Glucose Concentration at First-Stage on 2KGA Production

The optimal residual glucose concentration of the first-stage of fermentation, depending on the fermentation time, provides the activated cells with high densities without a lag phase for the following stage experiments (Ji et al., 2014). On the basis of the time course of 2KGA batch fermentation by *P. plecoglossicida* JUIM01, 80% of the cultivated broth (28 L) from the first-stage was transferred to second fermentor for the second-stage of fermentation at the point when the residual glucose concentration of the first-stage reached between 13.27 and 55.02 g/L (corresponding fermentation occurred from 8.0 to 13.0 h). Seven liters of feeding media containing glucose at 300.0 g/L and CaCO_3_ at 82.5 g/L were supplemented into the second-stage fermentor for the following fermentation.

The second-stage fermentation proceeded until the glucose was used up. The 2KGA fermentation performances were summarized in Table 2. 2KGA concentrations in the first-stage broth were 120.04, 109.70, 94.47, 83.51, and 74.94 g/L when the residual glucose had decreased to 13.27, 24.13, 36.38, 44.69, and 55.02 g/L (corresponding with fermentation times of 13.0, 11.5, 10.0, 9.0, and 8.0 h), respectively. Final cell concentrations in all of the experimental groups reached roughly 3.80 g/L in the broths. The 2KGA yield and productivity were at their highest levels of 0.9443 g/g and 9.51 g/(L⋅h), respectively, when the residual glucose concentration was 24.13 g/L (a fermentation time of 11.5 h).

**TABLE 2:** Effect of residual glucose concentration at first-stage on 2KGA production.

Parameters	Residual glucose concentration at first-stage (g/L)				
	
	13.27 ± 1.78	24.13 ± 3.78	36.38 ± 3.90	44.69 ± 4.36	55.02 ± 2.80
**First stage**					
Initial glucose (g/L)	140.0	140.0	140.0	140.0	140.0
Consumption of glucose (g/L)	126.73 ± 1.78	115.87 ± 3.78	103.62 ± 3.90	95.31 ± 4.36	84.98 ± 2.80
Initial cell concentration (g/L)	0.81 ± 0.08	0.79 ± 0.10	0.83 ± 0.08	0.81 ± 0.07	0.84 ± 0.04
Final cell concentration (g/L)	3.92 ± 0.04	3.83 ± 0.10	3.80 ± 0.04	3.78 ± 0.10	3.76 ± 0.08
Initial 2KGA (g/L)	0.30 ± 0.13	0.28 ± 0.03	0.27 ± 0.07	0.33 ± 0.06	0.31 ± 0.04
Final 2KGA (g/L)	120.04 ± 1.46	109.70 ± 3.48	94.97 ± 4.77	85.51 ± 3.62	74.94 ± 2.79
Produced 2KGA (g/L)	119.74 ± 1.58	109.42 ± 3.51	94.70 ± 4.84	85.18 ± 3.68	74.63 ± 2.83
2KGA yield (g/g)	0.9448 ± 0.0008	0.9443 ± 0.0005	0.9137 ± 0.0123	0.8938 ± 0.0023	0.8781 ± 0.0043
Fermentation time (h)	13.0	11.5	10.0	9.0	8.0
2KGA productivity (g/L⋅h)	9.21 ± 0.12	9.51 ± 0.31	9.47 ± 0.48	9.46 ± 0.41	9.33 ± 0.35
**Second stage**					
Glucose concentration after feeding media (g/L)	70.60 ± 1.40	79.23 ± 3.11	89.10 ± 3.13	95.74 ± 3.48	104.01 ± 2.26
2KGA after feeding media (g/L)	96.03 ± 1.16	87.76 ± 2.79	73.98 ± 3.81	66.81 ± 2.90	58.75 ± 2.22
Cell concentration after feeding media (g/L)	3.13 ± 0.03	3.06 ± 0.08	3.04 ± 0.04	3.02 ± 0.08	3.01 ± 0.07
Residual glucose concentration after fermentation (g/L)	0.14 ± 0.07	0.15 ± 0.07	0.21 ± 0.10	0.12 ± 0.06	0.17 ± 0.08
2KGA concentration after fermentation (g/L)	167.88 ± 1.00	169.10 ± 0.59	165.78 ± 0.13	165.02 ± 1.44	163.31 ± 0.87
Cell concentration after fermentation (g/L)	3.71 ± 0.08	3.90 ± 0.08	3.74 ± 0.06	3.81 ± 0.06	3.85 ± 0.07
Produced 2KGA at 2nd stage (g/L)	71.85 ± 2.16	81.34 ± 3.38	91.81 ± 3.94	98.21 ± 4.34	104.56 ± 3.09
2KGA yield at 2nd stage (g/g)	1.0176 ± 0.0104	1.0266 ± 0.0023	1.0303 ± 0.0080	1.0257 ± 0.0081	1.0052 ± 0.0079
Fermentation time at 2nd stage (h)	8.5	9.5	11.0	12.0	13.0
2KGA productivity at 2nd stage (g/L⋅h)	8.45 ± 0.25	8.56 ± 0.35	8.35 ± 0.36	8.18 ± 0.36	8.04 ± 0.24
In total*					
Total fermentation time (h)	21.5	21.0	21.0	21.0	21.0
Total 2KGA yield (g/g)	0.9760 ± 0.0058	0.9832 ± 0.0035	0.9638 ± 0.0007	0.9594 ± 0.0084	0.9494 ± 0.0051
Total 2KGA productivity (g/L⋅h)	7.81 ± 0.05	8.05 ± 0.03	7.89 ± 0.01	7.86 ± 0.07	7.78 ± 0.04

*, “In total” refers to the combination of first- and second- stage fermentation.

After transferring 80% of the cultivated broth (28 L) from the first-stage to the second-stage fermentor and feeding 7 L of fresh feeding media, the glucose concentration in the second-stage fermentor increased to 70.60, 79.23, 89.10, 95.74, and 104.01 g/L as 2KGA concentrations decreased to 96.03, 87.76, 73.98, 66.81, and 58.75 g/L, respectively, and the cell densities also decreased correspondingly to about 3.0 g/L. At the end of the second-stage fermentation, the maximum productivity achieved was 8.56 g/(L⋅h) with 81.34 g/L of 2KGA and a 2KGA yield of 1.0266 g/g.

Considering the fermentation performance of both the first- and second-stages, the highest productivity of 8.05 g/(L⋅h) and yield of 0.9832 g/g were obtained when the residual glucose concentration was 24.13 g/L. Hence, it could be concluded that the broth in the first-stage could transfer to the second-stage fermentor for further fermentation when the residual glucose concentration was about 25.0 g/L (a fermentation time of 11.5 h) providing the live cells and glucose for the next stage.

### Effect of Removing Broth Volumes of First-Stage on 2KGA Production

The cultivated broth in the first-stage contained live cells, residual glucose and produced 2KGA (Zhang et al., 2010). Removing volumes directly affected the remaining active cells, initial glucose level and 2KGA content for the next stage of fermentation. Accordingly, various broth volumes ranging from 50.0% to 80.0% (*v*/*v*) were taken after 11.5 h of first-stage fermentation, and transferred to second-stage fermentors to investigate the influence of removing volumes on 2KGA fermentation performance. As summarized in Table 3, after adding the corresponding volumes (50.0%, 60.0%, 70.0%, and 80.0%, *v*/*v*) of fresh media into each first-stage fermentor to bring the final working volume to 35 L, the glucose concentrations changed to 81.54, 92.04, 104.47, and 115.26 g/L, cell densities decreased to 1.85, 1.54, 1.18, and 0.82 g/L, and 2KGA concentrations changed to 55.72, 44.49, 33.10, and 21.91 g/L, respectively. At the end, 2KGA concentrations in all experiments reached to about 110.0 g/L. The maximum cell concentration of 3.90 g/L, 2KGA yield of 0.9564 g/g and productivity of 9.53 g/(L⋅h) were obtained by removing 70.0% (v/v) of the broth (adding 70.0%, v/v, of fresh media). The glucose concentrations in the second-stage fermentation ranged from 79.87 g/L to 160.47 g/L after transferring 80.0%, 70.0%, 60.0% and 50.0% (*v*/*v*) of first-stage broth and feeding corresponding volumes (20%, 30%, 40%, and 50%, *v*/*v*) of media containing 300.0 g/L of glucose. As a consequence, resulting 2KGA concentrations were 160.29, 134.84, 109.12, and 81.83 g/L, respectively. A Maximum yield of 1.0250 g/g was observed with a productivity of 8.39 g/(L⋅h) when removing volume of first-stage broth was 70.0% (*v*/*v*).

**TABLE 3:** Effect of removing broth volumes of first-stage on 2KGA production.

Parameters	V/V_1_ (%)			
	
	50.0	60.0	70.0	80.0
**First stage**				
Initial glucose (g/L)	81.54 ± 1.91	92.04 ± 1.48	104.47 ± 0.91	115.26 ± 0.76
Residual glucose (g/L)	23.94 ± 2.74	24.01 ± 2.25	24.79 ± 2.89	23.49 ± 2.69
Consumption of glucose (g/L)	57.60 ± 1.95	68.03 ± 3.00	79.68 ± 3.61	91.77 ± 2.83
Initial cell concentration (g/L)	1.85 ± 0.06	1.54 ± 0.04	1.18 ± 0.02	0.82 ± 0.04
Final cell concentration (g/L)	3.62 ± 0.04	3.82 ± 0.09	3.90 ± 0.03	4.08 ± 0.16
Initial 2KGA (g/L)	55.72 ± 1.06	44.49 ± 0.72	33.10 ± 0.65	21.91 ± 0.16
Final 2KGA (g/L)	110.50 ± 2.59	109.03 ± 2.06	109.29 ± 3.75	109.16 ± 3.29
Increased 2KGA (g/L)	54.79 ± 3.22	64.54 ± 2.23	76.20 ± 3.17	87.25 ± 3.13
2KGA yield (g/g)	0.9508 ± 0.0337	0.9491 ± 0.0164	0.9564 ± 0.0129	0.9507 ± 0.0077
Fermentation time (h)	6.00 ± 0.50	7.00 ± 0.35	8.00 ± 0.35	10.00 ± 0.50
2KGA productivity (g/L⋅h)	9.15 ± 0.36	9.23 ± 0.26	9.53 ± 0.23	8.73 ± 0.18
**Second stage**				
Initial glucose (g/L)	160.47 ± 2.88	133.02 ± 1.40	106.44 ± 2.01	79.87 ± 3.37
Residual glucose (g/L)	0.10 ± 0.05	0.18 ± 0.06	0.15 ± 0.03	0.08 ± 0.01
Initial cell concentration (g/L)	1.81 ± 0.03	2.29 ± 0.04	2.73 ± 0.04	3.26 ± 0.08
Final cell concentration (g/L)	3.73 ± 0.07	3.82 ± 0.06	3.88 ± 0.07	3.89 ± 0.04
Initial 2KGA (g/L)	54.05 ± 2.67	65.39 ± 1.73	75.56 ± 1.95	87.02 ± 3.86
Final 2KGA (g/L)	214.34 ± 1.96	200.22 ± 1.71	184.68 ± 1.92	168.85 ± 2.49
Produced 2KGA (g/L)	160.29 ± 4.63	134.83 ± 3.44	109.12 ± 3.87	81.83 ± 6.35
2KGA yield (g/g)	0.9987 ± 0.0109	1.0135 ± 0.0152	1.0250 ± 0.0171	1.0238 ± 0.0364
Fermentation time (h)	19.5	16.0	13.0	9.5
2KGA productivity (g/L⋅h)	8.22 ± 0.24	8.43 ± 0.21	8.39 ± 0.30	8.61 ± 0.67
In total*				
Total 2KGA yield (g/g)	0.9743 ± 0.0089	0.9815 ± 0.0084	0.9823 ± 0.0102	0.9817 ± 0.0144
Total fermentation time (h)	25.5	23.0	21.0	19.5
Total 2KGA productivity (g/L⋅h)	8.41 ± 0.08	8.71 ± 0.07	8.79 ± 0.09	8.66 ± 0.13

*, “In total” refers to the combination of first- and second- stage fermentation.

Combining the first- and second-stage of fermentation, removing 70.0% (*v*/*v*) of the volume from the first-stage fermentor could be selected for following study with a total productivity of 8.79 g/(L⋅h) and yield of 0.9823 g/g, indicating that first-stage fermentation could produce 2KGA at 76.20 g/L and provide a high density of *P. plecoglossicida* JUIM01 cells for highly efficient 2KGA fermentation at the second-stage.

### Effect of Feeding Glucose Concentration on Second- Stage Fermentation

A broth volume set at 70.0% (*v*/*v*) (24.5 L) was transferred into the second-stage fermentor, and 10.5 L of fresh media containing various glucose concentrations from 250.0 to 450.0 g/L were fed to investigate their effect on 2KGA fermentation performance. As presented in Table 4, after feeding various concentrations of glucose, the initial glucose concentrations in the second-stage fermentor changed with a range from 90.35 to 150.24 g/L with initial 2KGA concentrations of about 76.0 g/L. Increasing the glucose concentration in the feeding media directly prolonged the fermentation time from 11.5 to 19.0 h, and led to final cell concentrations with a range from 3.99 to 3.73 g/L and 2KGA concentrations from 168.72 g/L to 228.83 g/L. A maximum 2KGA yield of 0.9872 g/g and a higher productivity of 8.97 g/(L⋅h) were observed by feeding 10.5 L of media containing 400.0 g/L of glucose, which was attributed to the increased produced 2KGA concentration of 139.83 g/L. Hence, feeding media containing 400 g/L of glucose is suitable for second-stage fermentation due to it producing the highest yield, highest produced 2KGA concentration and highest productivity.

**TABLE 4:** Effect of feeding glucose concentration on second-stage fermentation.

Parameters	Glucose concentration in feeding media (g/L)				
	**250.0**	**300.0**	**350.0**	**400.0**	**450.0**

Initial glucose (g/L)	90.35 ± 2.11	105.42 ± 1.24	120.62 ± 1.99	135.63 ± 1.68	150.24 ± 2.28
Residual glucose (g/L)	0.14 ± 0.06	0.15 ± 0.06	0.16 ± 0.07	0.19 ± 0.05	0.21 ± 0.03
Initial cell concentration (g/L)	2.74 ± 0.04	2.73 ± 0.04	2.74 ± 0.04	2.74 ± 0.03	2.73 ± 0.03
Final cell concentration (g/L)	3.99 ± 0.09	3.91 ± 0.03	3.84 ± 0.06	3.80 ± 0.06	3.73 ± 0.06
Initial 2KGA (g/L)	76.47 ± 1.43	76.52 ± 1.61	75.88 ± 1.07	75.38 ± 1.82	76.97 ± 0.57
Final 2KGA (g/L)	168.72 ± 2.07	184.26 ± 1.29	199.19 ± 1.12	215.21 ± 1.93	228.84 ± 2.48
Increased 2KGA (g/L)	92.25 ± 3.25	107.74 ± 1.89	123.31 ± 1.59	139.83 ± 2.06	151.87 ± 2.51
2KGA yield (g/g)	1.0209 ± 0.0210	1.0221 ± 0.0149	1.0224 ± 0.0107	1.0309 ± 0.0097	1.0108 ± 0.0042
Fermentation time (h)	11.5	13.0	14.5	16.0	19.0
2KGA productivity (g/L⋅h)	8.02 ± 0.28	8.29 ± 0.15	8.50 ± 0.11	8.74 ± 0.13	7.99 ± 0.13
Total 2KGA yield (g/g)*	0.9753 ± 0.0109	0.9801 ± 0.0062	0.9812 ± 0.0050	0.9872 ± 0.0081	0.9821 ± 0.0097
Total fermentation time (h)*	19.5	21.0	22.5	24.0	27.0
Total 2KGA productivity (g/L⋅h)*	8.65 ± 0.11	8.77 ± 0.06	8.85 ± 0.05	8.97 ± 0.08	8.48 ± 0.09

*, “total” refers to the combination of first- and second- stage fermentation.

### 2KGA Two-Stage Fermentation Under Optimal Conditions

**FIGURE 3:**
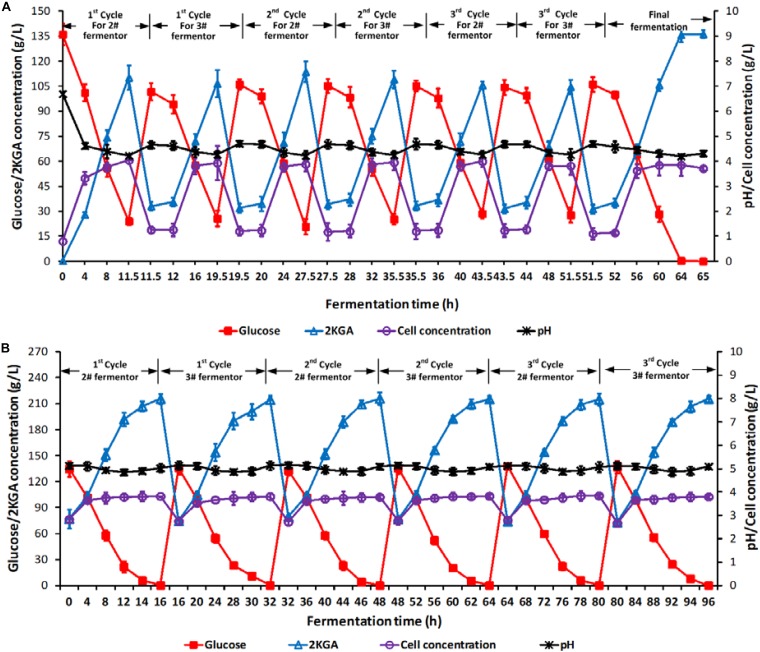
Time courses of 2KGA production, residual glucose concentration, cell concentration and pH in a 3-cycle two-stage semi-continuous fermentation. Time courses of **(A)** 1# fermentor for the first-stage fermentation and **(B)** 2# and 3# fermentors for the second-stage fermentation. Each cycle lasted for 32 h. At the end of first-stage fermentation, 70% (*v*/*v*) of broth (24.5 L) in 1# fermentor was taken out as the inoculum for second-stage fermentation (2# or 3# fermentor), which was started by feeding 10.5 L of 400.0 g/L of glucose. Data are the means of three independent experiments ± standard deviations (*n* = 3) analyzed in duplicate.

Two stage semi-continuous fermentation of 2KGA under the optimal conditions by *P. plecoglossicida* JUIM01 was verified for 3 cycles. Briefly, the 11.5-h cultured broth of 70% (*v*/*v*) at the first-stage (1# fermentor) was taken out and transferred into 2# fermentor at the second-stage. 10.5 L of fresh media containing glucose at 400.0 g/L was added into 2# fermentor at the second-stage for another 16-h cultivation until the residual glucose was consumed completely. At the same time, 24.5 L of fresh media containing glucose at 140.0 g/L, corn syrup powder at 7.5 g/L, urea at 0.15 g/L, KH_2_PO_4_ at 0.1 g/L and CaCO_3_ at 39.0 g/L was fed into the first-stage fermentor for a further 8-h cultivation with residual glucose of about 24.5 g/L, and 70.0% (*v*/*v*) of total broth volume was taken out and transferred to 3# fermentor at the second-stage. Similarly, 3# fermentor continued to ferment after feeding 10.5 L of fresh media containing glucose at 400.0 g/L until the residual glucose decreased to 0.1 g/L. Each of the two-stage semi-continuous cycles included one batch of first-stage fermentation (1# fermentor, cultivation time of 8.0 h) and one batch of second-stage fermentation (2# fermentor, fermentation time of 16.0 h), and one batch of first-stage fermentation (1# fermentor, cultivation time of 8.0 h) and one batch of second-stage fermentation (3# fermentor, fermentation time of 16.0 h). At the end of the 3rd cycle, all of the three fermentors would end with a residual glucose concentration of 0.1 g/L. Figure 3 presented the total time courses of the first- (A, 1# fermentor) and second-stage (B, 2# and 3# fermentors) fermentation of *P. plecoglossicida* JUIM01.

As shown in Figure 3A, after every taking out 70% of the cultivated broth from 1# fermentor at the first-stage and feeding fresh media, the initial glucose, 2KGA, and cell concentrations in 1# fermentor showed stable levels of approximately 105.0, 33.0, and 1.20 g/L, respectively. At the end of each fermentation, the glucose concentration decreased to about 25.0 g/L, cell density increased to 3.90 g/L and 2KGA concentration increased to 110.0 g/L. The stable 2KGA yield of 0.98 g/g and productivity of 9.5 g/(L⋅h) also indicated that the optimal removing volume of 70.0% (V/V_1_) could provide a stable cell density and residual glucose for second-stage fermentation.

The initial glucose, 2KGA and cell concentrations in 2# and 3# fermentor (at the second-stage) had comparable levels of 135.0, 75.0, and 2.80 g/L, respectively (Figure 3B). After 16.0 h of fermentation, glucose was completely consumed with a final 2KGA concentration of 215.0 g/L and cell concentration of 3.80 g/L. Accordingly, the 2KGA produced from of each run in each fermentor was 139.0 g/L with a total 2KGA yield of 0.988 g/g and productivity of 8.95 g/(L⋅h), which indicated that the second-stage fermentation under the optimal conditions had a high 2KGA production performance.

During 3-cycle fermentation, 25480.0 g of glucose in total was added in 1# fermentor at the first-stage and 21749.0 g glucose was consumed, resulting in 20703.0 g 2KGA produced with a total yield of 0.9519 g/g. Similarly, 28930.0 g of glucose in total was consumed in 2# and 3# fermentors at the second-stage, and 29302.0 g 2KGA was produced with an average 2KGA production of 215.40 g/L and a total yield of 1.0129 g/g. Hence, combining the first- and second- stages, a total of 50680.0 g of glucose was consumed, and 50005.20 g of 2KGA was produced with an average produced 2KGA concentration of 204.10 g/L and a total yield of 0.9867 g/g.

### Comparison of 2KGA Production by Different Culture Strategies

So far, four different fermentation processes including batch, fed-batch, semi-continuous and/or continuous fermentation have been used to produce 2KGA from glucose, cassava, RSH or corn starch hydrolyzate as substrates. 2KGA producing strains and their fermentation performance from this work and from literature reports were presented in Table 5. The 2KGA yields ranging from 0.92 g/g glucose to 1.08 g/g glucose were obtained using the continuous fermentation process by *S. marcescens* NRRL B-486 (Minisenhermer et al., 1965). Sun et al. (2014) developed a two-stage fermentation strategy using the first stage to maintain a neutral pH favoring cell growth and second stage which switched to acidic conditions favoring 2KGA accumulation, which produced a total of 186 g/L of 2KGA at 26 h with a conversion ratio of 0.98 mol/mol by *Klebsiella pneumoniae*. Our group had previously investigated 2KGA production strategies including batch, semi-continuous, continuous and even direct bioconversion of glucose using free resting cells of *P. fluorescens* AR4 (Sun et al., 2012, 2013, 2015). The strain *P. fluorescens* AR4 could convert 476.88 g/L of glucose into 444.96 g/L of 2KGA with a total productivity of 6.74 g/L and yield of 0.93 g/g during 4-run semi-continuous fermentation (Sun et al., 2012). At the steady state of continuous process, the amount of 2KGA was determined to be 135.92 g/L with 8.83 g/(L⋅h) average volumetric productivity and a 0.9510 g/g yield, and achieved a maximum 2KGA production of 195.0 g/L with 3.05 g/(L⋅h) total productivity and a 1.07 g/g yield during the 64-h direct bioconversion process. In this study, a two-stage semi-continuous fermentation of *P. plecoglossicida* JUIM01 consumed 50680.0 g of glucose and produced 50005.20 g of 2KGA with a total yield of 0.9867 g/g during a 3-cycle fermentation within 32 h. The average productivities of the first- and second- stages were calculated at 9.53 and 8.95 g/(L⋅h), respectively. Hence, two-stage semi-continuous fermentation inherited advantages such as high 2KGA production performance of semi-continuous process and avoided the lag time for cleaning, sterilization, and inoculation of each turnover. Additionally, the feeding media for the second-stage only consisted of glucose at a high concentration with no addition of either nitrogen or minerals, which would reduce the cost of industrial production.

**TABLE 5:** Comparison between the literature results cited and this work in 2KGA fermentation.

Organism	Raw material	Fermentation type	Total fermentation time (h)	2KGA production performance			Reference
				
				Concentration (g/L)	Productivity (g/L⋅h)	Yield (g/g)	
*Serratia marcescens* NRRL B-486	Glucose	Continuous	16∼40	/	/	0.92∼1.08	Minisenhermer et al., 1965
*Pseudomonas fluorescens* IFO 14808	Glucose	Batch	75	/	/	0.4	Tanimura et al., 2003
*Pseudomonas aeruginosa* IFO 3448	Cassava	Fed-batch	319	35	0.55	/	Chia et al., 2008
*Pseudomonas fluorescens* AR4	Rice starch	Semi-continuous	66	444.95	6.74	0.93	Sun et al., 2012
*Pseudomonas fluorescens* AR4	Corn starch hydrolyzate	Continuous	96	135.92	8.83 (steady state)	0.95	Sun et al., 2013
*Arthrobacter globiformis* C224	Rice starch hydrolyzate	Continuous	96∼120	124.74	11.23	0.97	Teng et al., 2013
*Klebsiella pneumoniae* ΔbudA	Glucose	Two-stage fed-batch	26	186	7.15	1.05	Sun et al., 2014
*Pseudomonas fluorescens* AR4	Glucose	Batch	64	195	3.05	1.07	Sun et al., 2015
*Gluconobacter oxidans*/pBBR-3510-ga2dh	Gluconic acid	Batch	45	486	10.08	1.01	Shi et al., 2015
*Gluconobacter oxydans*_*tufB*_*ga2dh*	Gluconic acid/glucose	Batch	45/18	453.3/321	10.07/17.83	0.94/1.19*	Li et al., 2016
*Pseudomonas plecoglossicida* JUIM01	Rice starch hydrolyzate/glucose	Two-stage semi-continuous	96	110.0 (first stage)/ 215.0 (second stage)	9.53 (first stage)/ 8.95 (second stage)	0.9867	In this work

* The yield of 1.19 g/g was calculated by 321 g/L 2KGA dividing 270 g/L of glucose, equivalent to a molar yield of 110.31%. However, the theoretical maximum yield from glucose to 2KGA should be no more than 1.08 g/g glucose. Although the yield reported by Li et al. (2016) is 99.1%, they didn’t explain how this number was calculated in their manuscript.

## Conclusion

A two-stage semi-continuous fermentation has been developed with the cooperation of 3 fermentors to enhance the 2KGA production performance by *P. plecoglossicida* JUIM01 using RSH as the substrate. A glucose concentration of 140.0 g/L was ideal for the first-stage fermentation due to its highest total 2KGA productivity of 7.58 g/(L⋅h) and cell concentration of 3.91 g/L. The optimal parameters for two-stage semi-continuous fermentation included 8.0-h fermentation time for the first-stage with a residual glucose concentration of about 25.0 g/L, 70.0% (*v*/*v*) of removing volume, and 400.0 g/L of feeding glucose for the second-stage fermentation. During 3-cycle fermentation, 50680.0 g of glucose was consumed, and 50005.20 g of 2KGA was obtained with a total yield of 0.9867 g/g by *P. plecoglossicida* JUIM01. In conclusion, the proposed two-stage semi-continuous fermentation showed a significant potential for industrial application based on its increased 2KGA production performance including concentration, yield and productivity from glucose in rice starch hydrolyzate, low input of feeding media, and decrease of processing period.

## Data Availability Statement

The raw data supporting the conclusions of this article will be made available by the authors, without undue reservation, to any qualified researcher.

## Author Contributions

W-JS and Z-HX conceived of the study and designed the experiments. LS and D-MW performed and analyzed results and drafted the manuscript. F-JC, J-SG, X-MZ, and J-SS performed partial experiments and analyzed results. All authors read and approved the manuscript.

## Conflict of Interest

W-JS and F-JC had coopereation with Parchn Sodium Isovitamin C Co. Ltd., by providing experimental materials including rice starch hydrolysate, and were/are not employed by company Parchn Sodium Isovitamin C Co. Ltd. The remaining authors declare that the research was conducted in the absence of any commercial or financial relationships that could be construed as a potential conflict of interest.
